# Clinical significance of the serum IgM and IgG to SARS‐CoV‐2 in coronavirus disease‐2019

**DOI:** 10.1002/jcla.23649

**Published:** 2020-11-13

**Authors:** Li‐xiang Wu, Hui Wang, Dan Gou, Gang Fu, Jing Wang, Bian‐qin Guo

**Affiliations:** ^1^ Department of Clinical Laboratory Chongqing Key Laboratory of Translational Research for Cancer Metastasis and Individualized Treatment Chongqing University Cancer Hospital & Chongqing Cancer Institute & Chongqing Cancer Hospital Chongqing China; ^2^ Department of Clinical Laboratory Chongqing Public Health Medical Treatment Center Chongqing China

**Keywords:** COVID‐19, IgG, IgM, SARS‐CoV‐2

## Abstract

**Objective:**

To explore the clinical value of serum IgM and IgG to SARS‐CoV‐2 in COVID‐19.

**Methods:**

105 COVID‐19 patients were enrolled as the disease group. 197 non‐COVID‐19 patients served as the control group. Magnetic chemiluminescent immunoassay (MCLIA) was used to detect the IgM and IgG.

**Results:**

The peak of positive rates of SARS‐CoV‐2 IgM was about 1 week earlier than that of IgG. It reached to peak within 15–21 days and then began a slowly decline. The positive rates of IgG were increased with the disease course and reached the peak between 22 and 39 days. The differences in sensitivity of the three detection modes (IgM, IgG, and IgM + IgG) were statistically significant. The largest group of test cases (illness onset 15–21 days) showed that the positive rate of IgG was higher than IgM. Also, the sensitivity of IgM combined with IgG was higher than IgM or IgG. IgM and IgG were monitored dynamically for 16 patients with COVID‐19, the results showed that serological transformation of IgM was carried out simultaneously with IgG in seven patients, which was earlier than IgG in four patients and later than IgG in five patients.

**Conclusion:**

The detection of SARS‐CoV‐2 IgM and IgG is very important to determine the course of COVID‐19. Nucleic acid detection combined with serum antibody of SARS‐CoV‐2 may be the best laboratory indicator for the diagnosis of SARS‐CoV‐2 infection and the phrase and predication for prognosis of COVID‐19.

## Graphical abstract

Antibody response to SARS‐CoV‐2 in 105 COVID‐19 patients. (A) The positive rate of SARS‐CoV‐2 antibody in 105 patients. (B) The changes of antibody titers to SARS‐CoV‐2 in 105 patients.

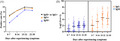

## 1 INTRODUCTION

Coronavirus disease‐2019 (COVID‐19) caused by Severe Acute Respiratory Syndrome Coronavirus 2 (SARS‐Cov‐2) is a severe infectious disease with high mortality. Because SARS‐Cov‐2 can be transmitted through the respiratory tract, early diagnosis is of great significance for cutting off the route of transmission.^1^, ^2^

The clinical guideline pointed out that the detection of SARS‐CoV‐2 RNA in upper and lower respiratory tract samples by reverse transcription polymerase chain reaction (RT‐PCR) is the gold standard for the diagnosis of COVID‐19.^3^, ^4^, ^5^ However, SARS‐CoV‐2 RNA testing based on throat or nasopharyngeal swabs yields frequent false‐negative. Many cases that were strongly epidemiological linked to SARS‐CoV‐2 exposure and with typical lung radiological findings remained RNA negative in their upper respiratory tract samples.^6^, ^7^ Therefore, it is of great necessary to use a rapid and accurate scheme of diagnosis based on different detection principles which could overcome the shortcomings of RNA detection.

SARS‐CoV‐2 antibody can be produced in COVID‐19 patients who were infected SARS‐CoV‐2 for 3–15 days.^8^ Therefore, antibody test of suspected COVID‐19 patients could be a good way that reduced missed diagnosis when RNA testing is negative. Lijia et al detected SARS‐CoV‐2 IgM and IgG antibodies in 15 COVID‐19 patients. They found that the shortest time was 1.5–2 days for the detectable antibody after symptom onset.^9^ Ma et al^10^ added the detection of SARS‐CoV‐2 IgA, which resulted a better diagnostic outcome in COVID‐19 early stages.

To further explore the value of application of SARS‐CoV‐2 IgM and IgG antibodies in the diagnosis and predication for course and prognosis of COVID‐19, we conducted a retrospective study that the level of SARS‐CoV‐2 IgM and IgG antibodies were tested in serial blood samples collected from 105 confirmed COVID‐19 patients and 197 non‐COVID‐19 patients using the MCLIA. And the level of IgM and IgG antibodies was dynamically monitored in 16 confirmed COVID‐19 patients to investigate the change of IgM and IgG with disease progress.

## 2 MATERIALS AND METHODS

### 2.1 Patients

105 cases of COVID‐19 were performed a retrospective study, diagnosed in Chongqing Public Health Medical Treatment Center from Jan 26 to Feb 21, 2020. According to the number of days from illness onset, patients in the disease group were divided into four groups to study the peak detection rates of IgM and IgG to SARS‐Cov‐2. Also, we focused on 16 cases from 105 cases of COVID‐19 to get the dynamic change of antibody concentration by collecting serum sample for different time points. 197 cases of non‐COVID‐19 served as the control group were collected between Feb 12, 2020, and Mar 30, 2020, from Chongqing University Cancer Hospital. The ethics have been approved by the Ethics Committee of Chongqing Public Health Medical Treatment Center and Chongqing University Cancer Hospital.

### 2.2 Detection of SARS‐CoV‐2 nucleic acids

Pharyngeal swab samples were collected and placed into a collection tube pre‐filled with 2ml virus preservation solutions. We purchased nucleic acid extraction and real‐time fluorescence quantitative PCR kit from Da'an Biotechnology Co., Ltd, and Sansure Biotechnology Co., Ltd for non‐COVID‐19 patients, respectively. The experimental processes were operated following the kit instructions.

### 2.3 Measurement of the IgG and IgM to SARS‐CoV‐2

Serum samples were collected using vacutainer tubes without anticoagulant, centrifuged after the blood completely coagulated, inactivated in a 56°C water bath for 45 min, and stored at −20°C. The SARS‐CoV‐2 IgM and IgG were tested by automatic chemiluminescence immunoassay analyzer, and the detection kit was provided by Bioscience. The antigens used in this kit were the nucleocapsid protein of SARS‐CoV‐2 and a peptide from spike protein of SARS‐CoV‐2 which were labeled with fluorescein isothiocyanate (FITC) and immobilized on the anti‐FITC antibody conjugated magnetic particles. Alkaline phosphatase conjugated with human IgG or IgM antibody was used as the detection antibody, and 3‐[2‐spiroadamatane]‐4‐methoxy‐4‐[3‐phosphoryloxy]‐phenyl‐1,2‐dioxetane) Dioxetane (AMPPD) was used as the substrate. Relative luminous unit (RLU) of each sample tube was positively correlated with the SARS‐CoV‐2 antibody titer. Sample value/cutoff values (S/CO) = the RLU of samples/the RLU of critical value. S/CO < 1.0, the test results were seen as negative, conversely, positive. Cutoff value of the detection kit was defined as: cutoff = mean value of positive control RLU × 0.1 + mean value of negative control RLU. The antibody level in the article was represented by log2 (S/CO + 1). All tests are conducted under strict biosafety conditions.

### 2.4 Statistical analysis

Statistical analysis was performed using SPSS software 22.0. Median (quartile) data were used for continuous variable, and Wilcoxon rank‐sum test was used for comparison between different groups. Countable data were expressed in percentage and analyzed by chi‐square test. Take *ɑ* equal to 0.05 as the inspection standard. *p* ≤ 0.05 was treated as a significant difference.

## 3 RESULTS

### 3.1 The demographic and characteristics of enrolled patients

There were 302 patients adopted in the present retrospective study, including 197 non‐COVID‐19 and 105 COVID‐19 patients. A total of 234 blood samples were used to detect antibody against SARS‐CoV‐2 for the former, whereas 152 for the latter. Non‐COVID‐19 patients had a median age of 54 years (IQR, 49–64), of whom 58.4% were female, and 44 years (IQR, 34–56) for COVID‐19 patients, 46.7% were female (Table 1).

**TABLE 1:** Demographic and clinical characteristics of enrolled patients

Characteristics	Non‐COVID‐19	COVID‐19	Total
Number	197	105	302
Age, Median (IQR)	54 (49,64)	44(34,56)	52(43,63)
Female	115 (58.4%)	49(46.7%)	164(54.3%)
Male	82(41.6%)	56(53.3%)	138(45.6)
Numbers of SARS‐CoV‐2 antibody tests detected per case, Median (IQR)	2(1–3)	2(1–3)	2(1–3)
Total number of test samples	234	152	386

Abbreviation: IQR, inter quartile range.

### 3.2 Positive rate of Specific IgM or IgG antibody against SARS‐CoV‐2 in non‐COVID‐19 and COVID‐19 patients

105 COVID‐19 patients were confirmed by testing SARS‐CoV‐2 nucleic acid of pharyngeal swabs using RT‐qPCR. 152 serum samples of 105 COVID‐19 were assigned to four groups according to collecting time after experiencing symptoms. There are 30 serum samples for IgM and IgG testing between 0 and 7 days after experiencing symptoms, 35 serum samples between 8 and 14 days, 51 serum samples between 15 and 21 days, 36 serum samples between 22 and 39 days (Table 2).

**TABLE 2:** Positive rate of IgM or IgG antibodies against SARS‐CoV‐2 in non‐COVID‐19 and COVID‐19 patients

Groups	*n*	IgM+ (%)	IgG+ (%)	IgM+ or IgG+ (%)
COVID‐19:0–7	30	36.67	63.33	66.67
COVID‐19:8–14	35	62.86	85.71	85.71
COVID‐19:15–21	51	88.24	94.12	100
COVID‐19:22–39	36	86.11	94.44	97.22
Non‐COVID‐19	197	2.03	6.60	8.12

**FIGURE 1:**
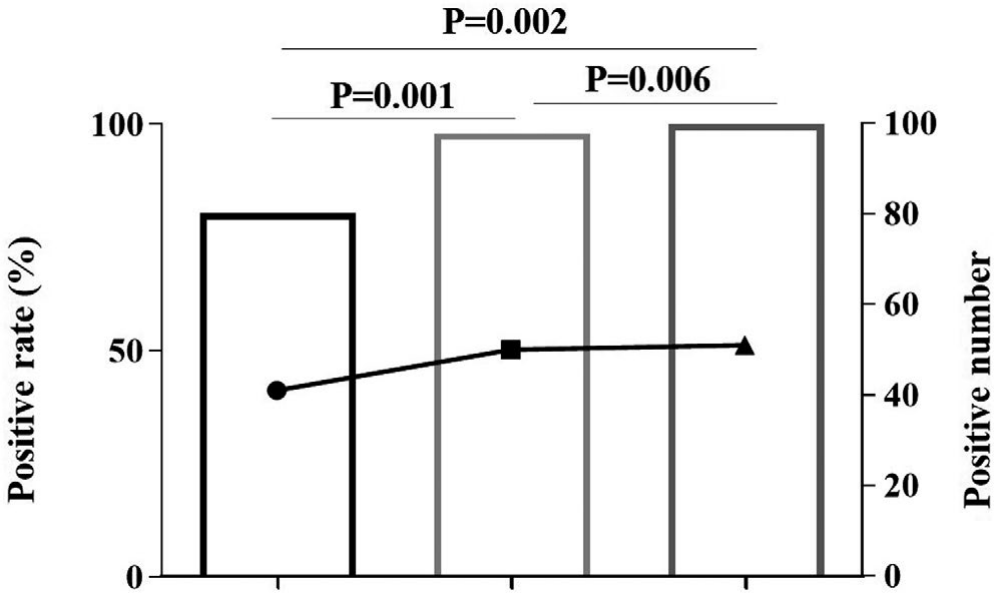
Positive rate of antibody to SARS‐CoV‐2 for the subgroup of 15–21 days after experiencing symptoms. The line graph represents positive number; the bar graph represents positive rate

We especially concerned the group which had the largest sample numbers collected between 15 and 21 days after experiencing symptoms. The positive rate of SARS‐CoV‐2 IgG antibody (94.12%, 48/51) was higher than IgM (88.24%, 45/51) (*χ* ^2^ = 10.129, *p* = 0.001); the combination analysis for IgM and IgG (100%, 51/51) higher than IgM (*χ* ^2^ = 10.896, *p* = 0.002) (Figure 1, Table 2).

We also analyzed the specificity of IgG and IgM antibodies in non‐COVID‐19 patients. There was no significant difference (*χ* ^2^ ＝ 3.340, *p* ＝ 0.921) between the IgM (97.97%, 193/197) and IgG (93.40%, 184/197). Also, the specificity of IgM or IgG was higher than the combination of IgM and IgG, but no significant difference (*χ* ^2^ ＝2.763, *p* ＝ 0.105; *χ* ^2^ ＝ 1.425, *p* ＝ 0.308).

### 3.3 The properties of SARS‐CoV‐2 IgM and IgG detected by MCLIA

To evaluate the properties of the detection kits for SARS‐CoV‐2 IgM and IgG by MCLIA, 105 COVID‐19 patients and 197 non‐COVID‐19 patients were tested (Table S1). The specificity of this kind of semi‐quantitative kits for SARS‐CoV‐2 IgM, IgG, and their combinations was 97.97%, 93.40%, and 91.88%, respectively. The sensitivity was 82.86%, 90.48%, and 96.16%, respectively (Table 3).

**TABLE 3:** The properties of SARS‐CoV‐2 IgM and IgG detected by MCLIA

	IgM (95% CI)	IgG (95% CI)	IgM or IgG (95% CI)
Sensitivity (%)	82.86% (75.65%,90.07%)	90.48% (84.86%,96.09%)	96.19% (92.53%,99.85%)
Specificity (%)	97.97% (96.00%,99.94%)	93.40% (89.93%,96.87%)	91.88% (88.06%,95.69%)
Accuracy (%)	92.72% (89.78%,95.65%)	92.38% (89.39%,95.38%)	93.38% (90.57%,96.18%)

Abbreviation: CI, confidence interval.

### 3.4 The change rule of IgM and IgG antibodies to SARS‐CoV‐2 in COVID‐19 patients

**FIGURE 2:**
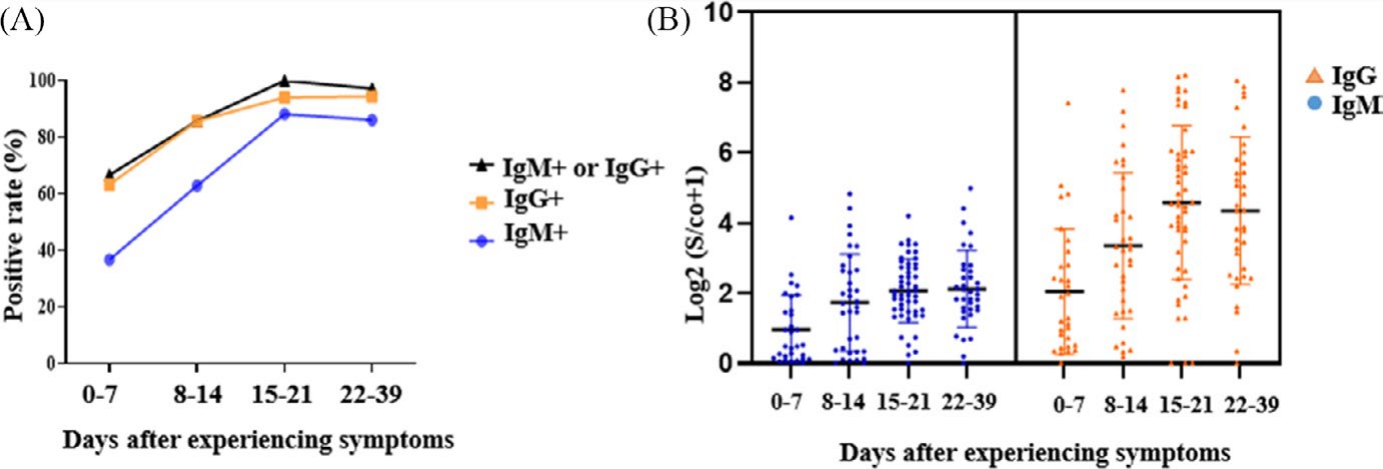
Antibody response to SARS‐CoV‐2 in 105 COVID‐19 patients. (A) The positive rate of SARS‐CoV‐2 antibody in 105 patients. (B) The changes of antibody titers in 105 patients

152 serum samples of 105 COVID‐19 patients were detected for IgM and IgG by MCLIA. The peak of positive rates of SARS‐CoV‐2 IgM and IgG was at different periods. The peak of positive rates of SARS‐CoV‐2 IgM (88.24%) was about 1 week earlier than that of IgG. The positive rates of SARS‐CoV‐2 IgM reached to peak within 15–21 days and then began to a slowly decline. The positive rates of IgG were increased with the disease period and reached the peak (94.44%) between 22 and 39 days (Figure 2A).

After approximately 0–7 days of experiencing symptoms, the titer of IgM and IgG antibodies gradually increased and began to decrease after 3 weeks (Figure 2B).

### 3.5 The seroconversion mode of IgM and IgG to SARS‐CoV‐2 in 16 COVID‐19 patients

**FIGURE 3:**
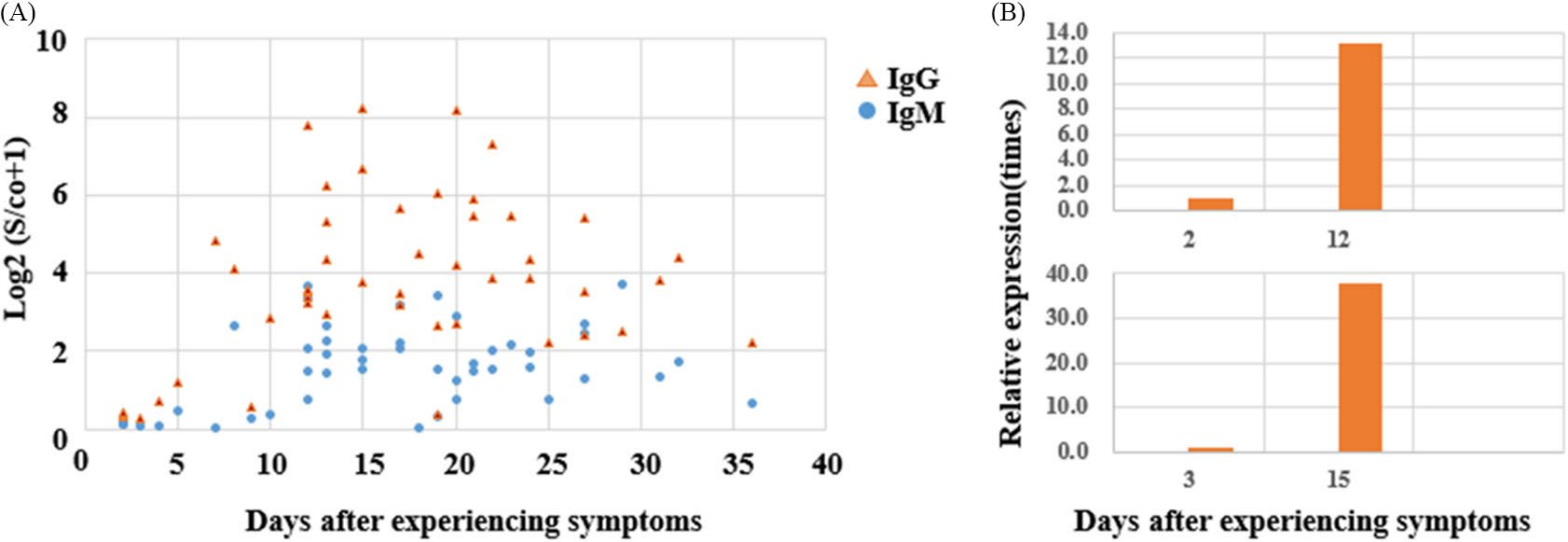
The changes of IgM and IgG to SARS‐CoV‐2 in serum during the course of 16 COVID‐19 patients. (A) The titers of IgM and IgG with the days after experiencing symptoms. (B) The titers comparison of IgG during the course of 16 COVID‐19 patients (only two patients depicted in Figure 3Band seeingAppendix S2for details)

SARS‐CoV‐2 IgM and IgG were monitored dramatically in 16 COVID‐19 with a median age of 46 years (IQR 38, 58) and consisted of nine women (56.3%). Fever and cough were the most common symptoms accounted for 87.5% and 43.8%, respectively (Table S2). Fig 3A described that the titters of IgM and IgG during the course of the disease were low within 0–10 days from the onset of experiencing symptoms. But the IgG titter was obviously more than IgM between 10 and 25 days after symptoms appear. Figure 3B depicted that IgG titter for serum samples collected at some points later was almost four times higher than the first samples in 10 COVID‐19 patients (see Appendix S2 for details). Just as recommended by china national health commission, a 4‐fold increase in the IgG titer can be used to confirm the infection of SARS‐CoV‐2 for suspected COVID‐19 patients. Therefore, dynamically monitoring SARS‐CoV‐2 antibody may be useful to help the diagnosis of COVID‐19.

**FIGURE 4:**
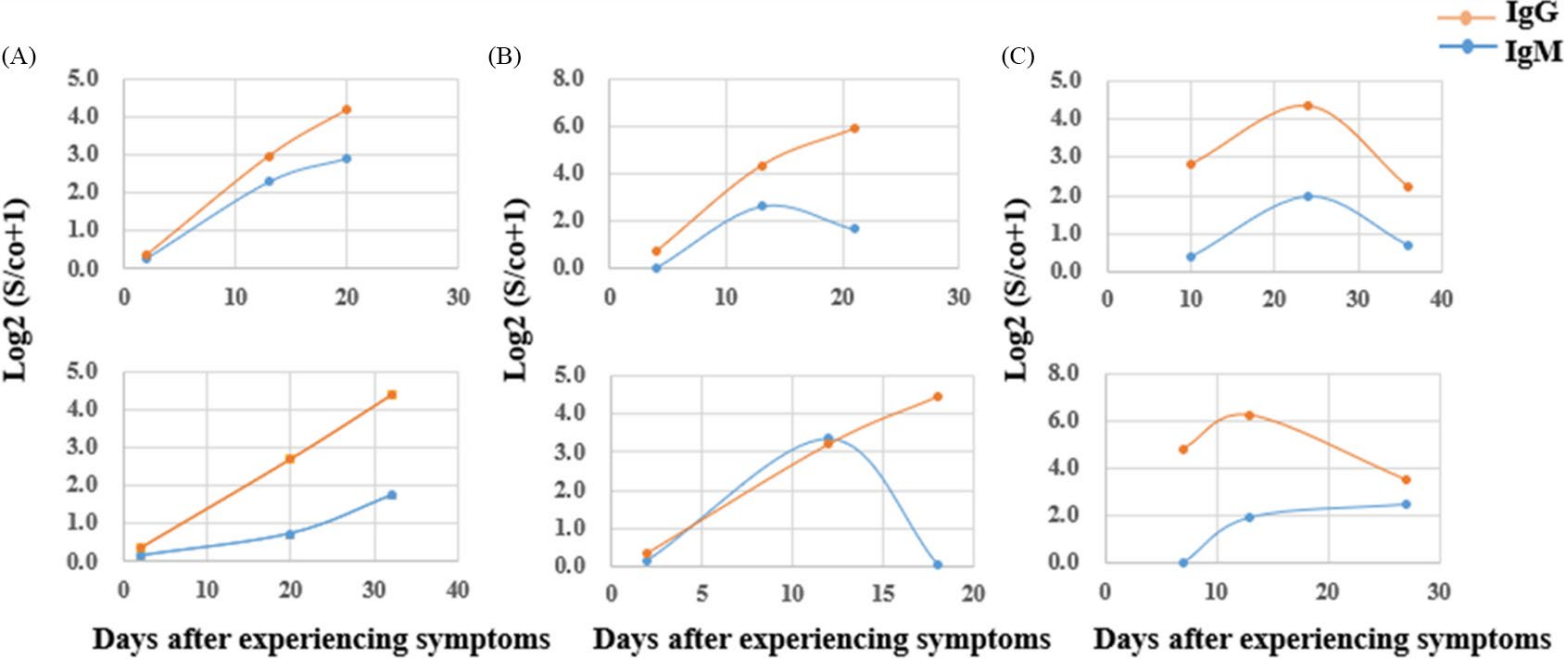
The three modes of seroconversion of IgM and IgG during the course of 16 COVID‐19 patients. (A) IgM and IgG antibodies changed in synchrony. (B) The IgM changed earlier than IgG antibody. (C) The IgG changedlater than IgM

Serological transformation of IgM and IgG in 16 COVID‐19 patients could be divided into three modes (Figure 4). It was showed that serological transformation of IgM was carried out simultaneously with IgG in seven patients, earlier than IgG ((Figure 4B) in four patients and later than IgG in five patients (Figure 4C). The three modes are consistent with the research papers published by Long et al.^11^

### 3.6 The analysis of positive rate of nucleic acid and SARS‐CoV‐2 antibody in 16 COVID‐19 patients

**FIGURE 5:**
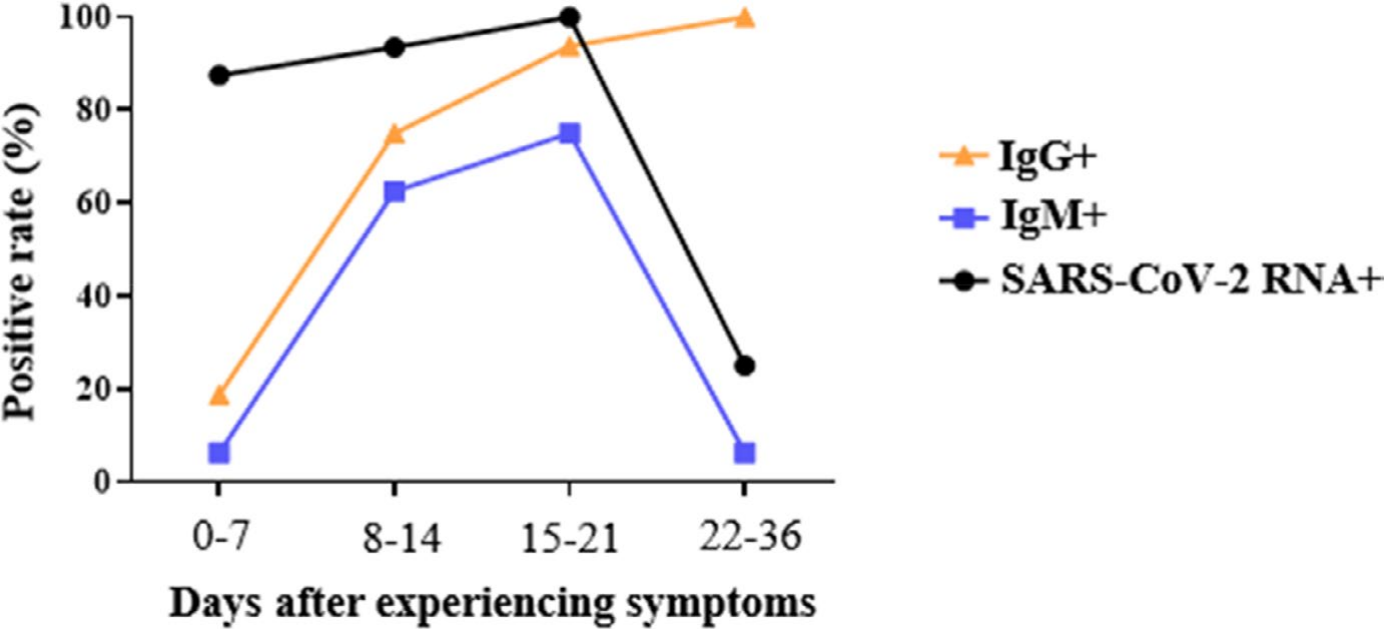
Positive rate of nucleic acid and antibody to SARS‐CoV‐2 in 16 COVID‐19 patients

Both nucleic acid and antibody to SARS‐CoV‐2 were dynamically monitored in 16 COVID‐19 patients, the percentage of IgM‐positive and RNA‐positive patients reached the highest coincidence between 15 and 21 days after the onset of symptoms and began to decline simultaneously. The percentage of IgG‐positive increased gradually over time within 36 days of symptoms and reached 100% between 22 and 36 days (Figure 5).

## 4 DISCUSSION

After the detection of IgM and IgG to SARS‐CoV‐2 becoming “golden standard” for the diagnosis of suspected COVID‐19 patients, The National Medical Products Administration of China approved urgently five antibody kits based on different principle of detection, some of them are used to test total antibody to SARS‐CoV‐2, the others to detect of IgM and IgG, seperatedly. Some of the kits are for qualitative such as colloidal gold test, and the others are quantitative such as ELISA or chemiluminescent immunoassay.^12^, ^13^, ^14^, ^15^ In this study, we carried out the semi‐quantitative detection of IgM and IgG to SARS‐CoV‐2 in 197 serum samples from non‐COVID‐19 patients by MCLIA to study specificity. Of the 197 non‐COVID‐19 patients including 234 serum samples (Table S3), 13 cases of SARS‐CoV‐2 IgG were false‐positive, five of which had S/CO value at the threshold, and the remaining eight patients were above the threshold (Figure S1A). There were four false positive of SARS‐CoV‐2 IgM. The S/ CO value of two cases was near the critical value, and the others was slightly higher than the critical value (Figure S1B). In addition to throat swab nucleic acid test, SARS‐CoV‐2 nucleic acid in serum samples of seven patients which had higher titer for IgM or IgG was tested, and the results were all negative. None of seven patients which were positive for IgM or IgG had symptoms of COVID‐19, and no COVID‐19 case had been appeared in Chongqing University Cancer hospital where the seven patients had been visiting until Apr 20, 2020 (Table S4). In order to further explore the cause of false‐positive, IgM and IgG antibodies of influenza virus, parainfluenza virus, Mycoplasma pneumonia, Chlamydia pneumonia, respiratory syncytial virus, adenovirus, and coxsackie virus were detected in seven serum samples, respectively, Also, rheumatoid factors (RF) and complement C1q were tested. There was no significant difference for above items between antibody positive groups and negative groups in non‐COVID‐19 patients (Figure S2). We also reviewed the medical records of these seven patients retrospectively, and these patients were also not treated with antibody preparations. We have not yet identified the reasons for false‐positive IgG or IgM antibodies in seven non‐COVID‐19 patients. Our test results were basically consistent with the specificity given in the reagent instructions (Figure S3). Taken 93.4% confidence interval as the standard for setting the reference interval according to the reagent specification, which means that 6.6% of the population is still not within the current reference interval, which may be one of the reasons for false positives. In general, the diagnose specificity of this kind of semi‐quantitative kits for SARS‐CoV‐2 IgM, IgG, and their combinations was 97.97%, 93.40%, and 91.88%, respectively. The diagnose sensitivity was 82.86%, 90.48% and 96.19%, respectively. Hu et al^16^ proposed heat‐treated serum samples could lead to false‐negative results of these samples. But this problem is not obvious in our research, and the sensitivity is acceptable. As reported in the literature, SARS‐CoV‐2 has four major structural proteins (spike protein, nucleocapsid protein, envelope protein and membrane protein) and some accessory open reading frame proteins.^17^, ^18^ When using different antigens to measure SARS‐CoV‐2 antibody, the results may be different. In this study, the antigens used in this kit were the nucleocapsid protein of SARS‐CoV‐2 and a peptide from spike protein of SARS‐CoV‐2. Compared with the study of Ma et al^10^ who used receptor‐binding domain of spike protein of SARS‐CoV‐2 as antigen, the diagnose specificity for SARS‐CoV‐2 IgG and IgM were both 96.8%, and the diagnose sensitivity was 92.3%, 99.8%. According to the literature, SARS‐CoV‐2 IgA was also elevated in COVID‐19 patients, the diagnose specificity and sensitivity of SARS‐CoV‐2 IgA could be reached to 98.1% and 98.6%.^10^ For this reason, the next work for us is to further evaluate the diagnostic value of IgA to ensure the integrity for this study.

Meanwhile, we analyzed the serum antibody results of 105 patients with COVID‐19 confirmed by nucleic acid test, and found it was great possibe that antibody detection was negative within 0–5 days for patients infected with SARS‐CoV‐2 virus, IgM liter would increase significantly after 1 week, and IgG liter reached a peak within 15‐21 days. Therefore, IgM and IgG to SARS‐CoV‐2 which were negative could not exclude the possibility of SARS‐CoV‐2 infection. The patient could be in the initial stage of infection. At this stage, IgM and IgG had not been produced or the titer was too low, resulting in false‐negative results of antibody. It is recommended that the samples should be collected again 3–5 days later and tested to determine whether there is a transformation of serological positive or significant increasing of antibody titer compared with the last collected samples.

During dynamic monitoring of antibody for 16 patients with COVID‐19, it was found that the serological transformation of IgM and IgG antibodies in the samples could be divided into three modes: The first was the simultaneous transformation of the two; the second was that the transformation of IgM in serum was earlier than IgG; the third was that the transformation of IgG in serum was earlier than IgM. The seroconversion patterns of antibody observed were consistent with the results of Long et al.^11^ According to the relationship between the concentrations of two antibodies in serum, we could know the course of disease and further guide the clinical treatment.

We also analyzed the positive rate of nucleic acid and antibody to SARS‐CoV‐2 of 16 patients and found that the positive rate of IgM antibody and nucleic acid had a synchronous change trend. The novel coronavirus pneumonia diagnosis and treatment plan (Trial Seventh Edition) of China mentioned that only patients with twice negative of throat swab nucleic acid testing can be discharged. The risk of infection of pharyngeal swabs is much greater than that of blood. Our results suggest that pharyngeal swabs could be collected when IgM turned negative, reducing the chance of occupational exposure. This conclusion is currently only used as a reference. We need more data to verify this result.

It has obvious advantages for the detection of IgM and IgG to SARS‐CoV‐2. Firstly, it is easy to collect serum samples and control the quality of samples. It could effectively avoid leak detection that the false‐negative results of nucleic acid which caused by the collection of samples or the lower viral load in a certain period of the disease. IgM and IgG to SARS‐CoV‐2 combined with nucleic acid detection could effectively avoid missed diagnosis of COVID‐19; Secondly, antibody testing might play an important role in evaluating the course of disease and predicting the prognosis according to the content of IgG and IgM; Thirdly, it is of major importance to the epidemiological investigation that some asymptomatic populations with recessive infection who have lived in the epidemic area or had closely contact with the patients subject to the dynamically detection of antibody; Fourthly, serological testing is advantageous with faster turn‐around time, high‐throughput, and less workload. Also, it could be widely developed in the basic laboratory where nucleic acid detection is carried out unconditionally. Finally, the detection of IgG could be used as one of the standards to evaluate whether patients with COVID‐19 who had recovered could donate the therapeutic plasma.

## CONFLICT OF INTEREST

The authors are declaring that there are no conflicts of interest.

## Supporting information

Appendix S1

Appendix S2

## Data Availability

All data included in this study are availability upon request by contact with the corresponding author.
