# Supplementary material for: Clinical significance of the serum IgM and IgG to SARS‐CoV‐2 in coronavirus disease‐2019
Source: J Clin Lab Anal. 2020 Nov 13;35(1):e23649. doi: 10.1002/jcla.23649 (PMC7843265; doi:10.1002/jcla.23649)
Supplement: Supplementary file 1 — Appendix S1 [file JCLA-35-e23649-s001.docx]

**Appendix S1**

**Clinical significance of the serum IgM and IgG in Coronavirus Disease 2019**

Li-xiang Wu^1, #^, Hui Wang^1, #^, Dan Gou^1, #^, Gang Fu^1^, Jing Wang ^2^, Bian-qin Guo^1, *^

1 Department of Clinical Laboratory, Chongqing Key Laboratory of Translational Research for Cancer Metastasis and Individualized Treatment, Chongqing University Cancer Hospital & Chongqing Cancer Institute & Chongqing Cancer Hospital, Chongqing, 400030, P.R. China

2 Department of Clinical Laboratory, Chongqing Public Health Medical Treatment Center, Chongqing, 400030, P.R. China

^#^ Contributed equally. Li-xiang Wu, Hui Wang and Dan Gou contributed equally to this work and are co-first authors.

^*^ Correspondence author: Bian-qin Guo, Department of Clinical Laboratory, Chongqing University Cancer Hospital, No. 181, Han Yu Road, Sha-ping-ba District, Chongqing 400030, P.R. China. E-mail address: [178098941@qq.com](mailto:178098941@qq.com)

Table S1 The results of SARS-CoV-2 IgM and IgG in COVID-19 and Non-COVID-19

| Groups |  | **IgM** | | **IgG** | | **IgM or IgG** | |
| --- | --- | --- | --- | --- | --- | --- | --- |
|  | Cases | Positive | Negative | Positive | Negative | Positive | Negative |
| COVID-19 | 105 | 87 | 18 | 95 | 10 | 101 | 4 |
| Non-COVID-19 | 197 | 4 | 193 | 13 | 184 | 16 | 181 |
| **Cases** | **302** | **91** | **211** | **108** | **194** | **117** | **185** |

Table S2. Demographic and baseline characteristics of 16 dynamically monitored patients.

| **Characteristics** | **n** |
| --- | --- |
| Age, Median (IQR) | 46(38,58) |
| Female (%) | 9(56.3) |
| Male (%) | 7(43.7) |
| Days between nucleic acids confirmed and experiencing symptoms, Median (IQR) | 2(1,5) |
| Days between nucleic acids turned negative and experiencing symptoms, Median (IQR) | 28.5(26.25,36.75) |
| Days between the first antibody test and experiencing symptoms, Median (IQR) | 17(12,22) |
| Days between the first antibody test and nucleic acids confirmed, Median (IQR) | 12(6.25,18) |
| Fever (%) | 14/16(87.5) |
| Cough (%) | 7/16(43.8) |
| Fatigue (%) | 4/16(25.0) |
| Runny nose scores (%) | 3/16(18.8) |
| Diarrhea (%) | 3/16(18.8) |
| Dizzy (%) | 2/16(12.5) |
| Abdominal pain (%) | 2/16(12.5) |

Table S3. Demographic characteristics of 197 enrolled patients.

| **Characteristics Total (N=197)** |
| --- |
| Age, Median (IQR) 54(49,64) |
| Female (%) 115(58.4) |
| Male (%) 82(41.6) |
|  |


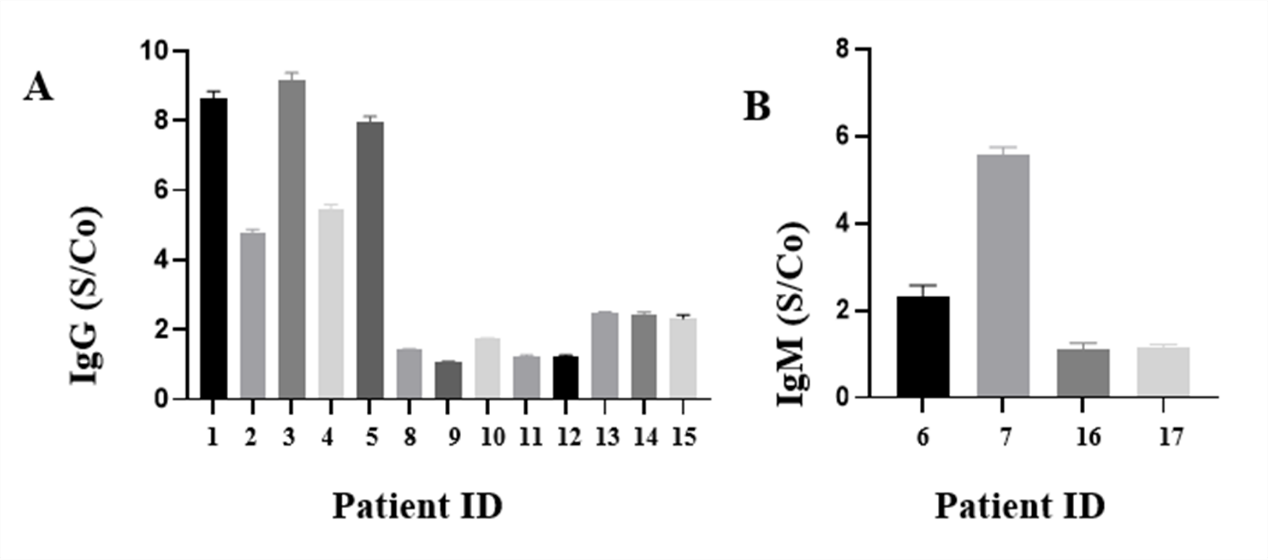


Figure S1. False-positive results of testing for antibody to COVID-19.

(A) False positive IgG test results in enrolled patients. IgG titer of 13 patients were greater than 1.00. (B) False positive IgM test results in enrolled patients. IgM titer of 4 patients were greater than 1.00.

Table S4. The follow-up time of COVID-19 antibodies false positive patients.

| Patient ID | Time of admission | Time of admission (Days) | Length of follow-up (Days) |
| --- | --- | --- | --- |
| 1 | 06/02/2020 | 24 | 60 |
| 2 | 24/02/2020 | 2 | 42 |
| 3 | 24/02/2020 | 24 | 42 |
| 4 | 07/03/2020 | 9 | 30 |
| 5 | 21/03/2020 | 4 | 16 |
| 6 | 10/02/2020 | 3 | 56 |
| 7 | 03/03/2020 | 9 | 33 |


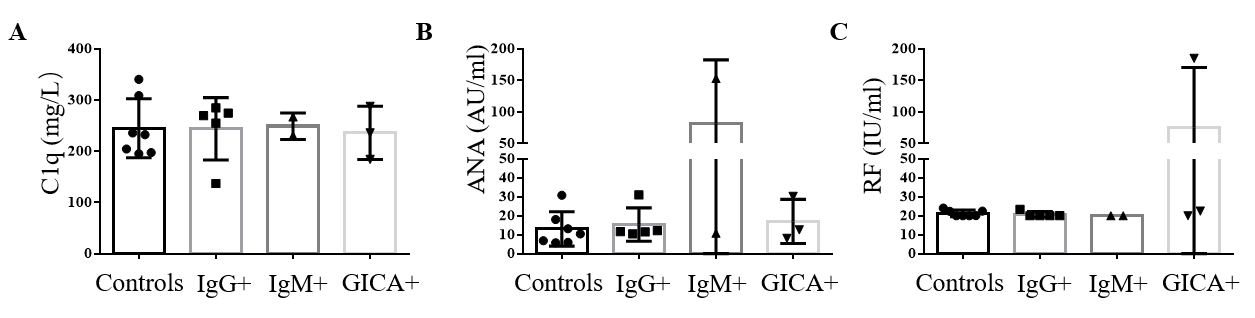


Figure S2. Laboratory findings of the control group and false positive groups. (A) C1q, (B) ANA, (C) RF. ANA=antinuclear antibody; RF=rheumatoid factor.


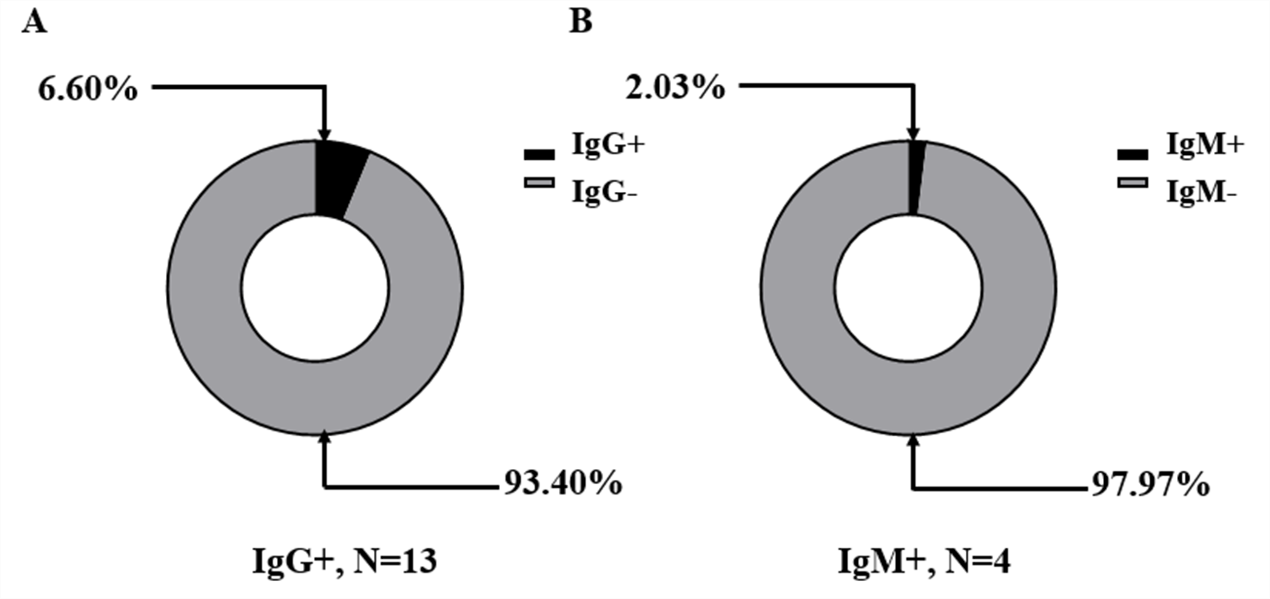


Figure S3. False-positive rate of CMIA and GICA assays detecting COVID-19 antibody. (A) IgG detection using CMIA assay in patients that were 6.6% positive. (B) IgM detection using CMIA assay in patients that were 2.03% positive. CMIA=Chemiluminescence microparticle immunoassays. GICA=the colloidal gold immunochromatography assay
