# Supplementary material for: Clinical significance of the serum IgM and IgG to SARS‐CoV‐2 in coronavirus disease‐2019
Source: J Clin Lab Anal. 2020 Nov 13;35(1):e23649. doi: 10.1002/jcla.23649 (PMC7843265; doi:10.1002/jcla.23649)
Supplement: Supplementary file 2 — Appendix S2 [file JCLA-35-e23649-s002.docx]

**Appendix S2**

| **Monitoring dramatically the changes of IgG against to SARS-CoV-2 in 16 COVID-19 patients** | | | | | |
| --- | --- | --- | --- | --- | --- |
|  | Patient ID | date of symptom onset | detected date | measured value of IgG（S/CO） | 4-fold higher for the latter than first time |
| 1 | 74907 | 2020/1/23 | 2020/1/28 | 1.31 | yes |
|  |  |  | 2020/2/9 | 49.03 |  |
|  |  |  | 2020/2/15 | 42.90 |  |
| 2 | 74910 | 2020/1/25 | 2020/1/28 | 0.23 | yes |
|  |  |  | 2020/2/9 | 100.62 |  |
|  |  |  | 2020/2/16 | 155.84 |  |
| 3 | 74923 | 2020/1/27 | 2020/1/29 | 0.36 | yes |
|  |  |  | 2020/2/9 | 38.23 |  |
|  |  |  | 2020/2/15 | 65.04 |  |
| 4 | 74952 | 2020/1/28 | 2020/1/30 | 0.27 | yes |
|  |  |  | 2020/2/9 | 8.37 |  |
|  |  |  | 2020/2/15 | 21.34 |  |
| 5 | 74962 | 2020/1/20 | 2020/1/30 | 6.08 | No |
|  |  |  | 2020/2/13 | 19.67 |  |
|  |  |  | 2020/2/25 | 3.67 |  |
| 6 | 74967 | 2020/1/21 | 2020/1/30 | 0.51 | yes |
|  |  |  | 2020/2/7 | 8.04 |  |
|  |  |  | 2020/2/17 | 4.39 |  |
| 7 | 74970 | 2020/1/28 | 2020/1/30 | 0.31 | yes |
|  |  |  | 2020/2/17 | 5.57 |  |
|  |  |  | 2020/2/29 | 19.84 |  |
| 8 | 74977 | 2020/1/27 | 2020/1/31 | 0.67 | yes |
|  |  |  | 2020/2/9 | 19.34 |  |
|  |  |  | 2020/2/17 | 59.22 |  |
| 9 | 74986 | 2020/1/29 | 2020/2/10 | 9.41 | No |
|  |  |  | 2020/2/17 | 5.25 |  |
|  |  |  | 2020/2/27 | 4.73 |  |
| 10 | 74987 | 2020/1/25 | 2020/2/6 | 219.41 | No |
|  |  |  | 2020/2/9 | 296.93 |  |
|  |  |  | 2020/2/14 | 287.17 |  |
| 11 | 75004 | 2020/1/27 | 2020/2/8 | 10.58 | Yes |
|  |  |  | 2020/2/17 | 42.94 |  |
|  |  |  | 2020/2/23 | 42.09 |  |
| 12 | 75006 | 2020/1/29 | 2020/2/5 | 27.63 | No |
|  |  |  | 2020/2/11 | 74.60 |  |
|  |  |  | 2020/2/25 | 10.57 |  |
| 13 | 75017 | 2020/1/26 | 2020/2/7 | 10.97 | No |
|  |  |  | 2020/2/12 | 10.27 |  |
|  |  |  | 2020/2/17 | 13.80 |  |
| 14 | 75147 | 2020/1/27 | 2020/2/5 | 0.31 | yes |
|  |  |  | 2020/2/11 | 3.64 |  |
|  |  |  | 2020/2/17 | 13.02 |  |
| 15 | 75161 | 2020/1/28 | 2020/2/5 | 16.24 | No |
|  |  |  | 2020/2/12 | 12.78 |  |
|  |  |  | 2020/2/21 | 13.47 |  |
| 16 | 75166 | 2020/2/3 | 2020/2/5 | 0.28 | yes |
|  |  |  | 2020/2/16 | 6.80 |  |
|  |  |  | 2020/2/23 | 17.21 |  |
